# Energy Harvested and Cooperative Enabled Efficient Routing Protocol (EHCRP) for IoT-WBAN

**DOI:** 10.3390/s20216267

**Published:** 2020-11-03

**Authors:** Muhammad Dawood Khan, Zahid Ullah, Arshad Ahmad, Bashir Hayat, Ahmad Almogren, Kyong Hoon Kim, Muhammad Ilyas, Muhammad Ali

**Affiliations:** 1 Centre of Excellence in IT, Institute of Management Sciences, Peshawar 25000, Pakistan; ms171606894@imsciences.edu.pk (M.D.K.); zahid.ullah@imsciences.edu.pk (Z.U.); bashir.hayat@imsciences.edu.pk (B.H.); ms171606966@imsciences.edu.pk (M.I.); muhammad.ali@imsciences.edu.pk (M.A.); 2 Department of IT and Computer Science, Pak-Austria Fachhochschule: Institute of Applied Sciences and Technology, Haripur 22620, Pakistan; yaarshad@gmail.com; 3 Department of Computer Science, College of Computer and Information Sciences, King Saud University, Riyadh 11451, Saudi Arabia; 4 School of Computer Science and Engineering, Kyungpook National University, Daegu 41566, Korea; kyong.kim@knu.ac.kr

**Keywords:** cooperative effort, energy harvesting, efficient data transmission, IoT, routing protocol, WBAN

## Abstract

The health industry is one of the most auspicious domains for the application of Internet of Things (IoT) based technologies. Lots of studies have been carried out in the health industry field to minimize the use of resources and increase the efficiency. The use of IoT combined with other technologies has brought quality advancement in the health sector at minimum expense. One such technology is the use of wireless body area networks (WBANs), which will help patients incredibly in the future and will make them more productive because there will be no need for staying at home or a hospital for a long time. WBANs and IoT have an integrated future as WBANs, like any IoT application, are a collection of heterogeneous sensor-based devices. For the better amalgamation of the IoT and WBANs, several hindrances blocking their integration need to be addressed. One such problem is the efficient routing of data in limited resource sensor nodes (SNs) in WBANs. To solve this and other problems, such as transmission of duplicate sensed data, limited network lifetime, etc., energy harvested and cooperative-enabled efficient routing protocol (EHCRP) for IoT-WBANs is proposed. The proposed protocol considers multiple parameters of WBANs for efficient routing such as residual energy of SNs, number of hops towards the sink, node congestion levels, signal-to-noise ratio (SNR) and available network bandwidth. A path cost estimation function is calculated to select forwarder node using these parameters. Due to the efficient use of the path-cost estimation process, the proposed mechanism achieves efficient and effective multi-hop routing of data and improves the reliability and efficiency of data transmission over the network. After extensive simulations, the achieved results of the proposed protocol are compared with state-of-the-art techniques, i.e., E-HARP, EB-MADM, PCRP and EERP. The results show significant improvement in network lifetime, network throughout, and end-to-end delay.

## 1. Introduction

The Internet of Things (IoT) and WBAN have an integrated future [1,2]. The IoT is one of the most modern promising research areas and is becoming a common platform where smart objects stay connected to the Internet for transmitting and receiving smart data [3,4,5]. The world is embracing the adoption of 5G while the 6G protocol [6,7,8], which is the next generation of advancement in wireless technology, is around the corner that will be a much great enhancer of IoT-based technologies. The IoT continues to grow as more and more devices are connected to it. General estimates reveal that the number of smart IoT devices will approach over 50 billion by 2022. The IoT landscape is evolving ever rapidly than before and is impacting the global economy. The Gartner Report sees its advantages in terms of economic impacts in 2022 to be around 2 trillion USD [9].

**Figure 1:**
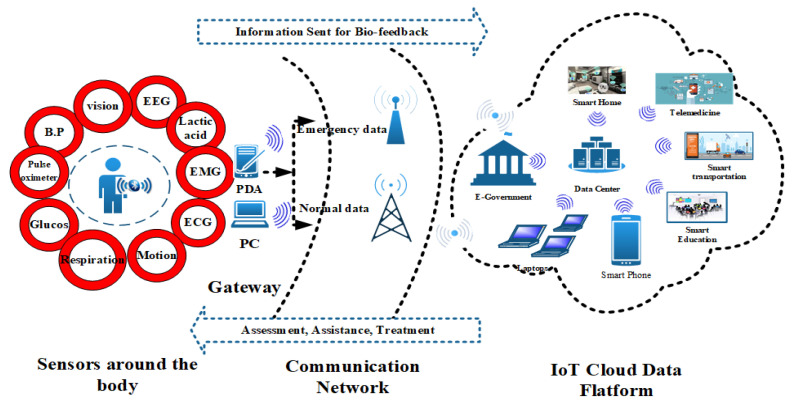
IoT-WBAN Communication structure.

Wireless body area networks (WBANs) are one of the most focused type of wireless sensor network (WSN) [10], in which communication takes place between individuals and machines through wireless technologies. A WBAN is defined as “using tiny sensors, in, on or around the human body for medical or any other purposes.” [11,12,13]. A WBAN may be used easily with IoT-based technology for monitoring of human body 24/7 [14]. In the healthcare domain, a properly established WBAN coupled with modern IoT-based devices has the potential to inform doctors in advance about the condition and criticality of the patient leading to improved quality of life. Heterogeneous IoT WBANs will bring revolutionary changes in healthcare in the future and a wide range of research areas as shown in Figure 1. It is believed that this technology will bring green changes in the field of medical. The required medical data is sensed by tiny low power sensors and forwarded to sink nodes where doctors take the necessary actions based on sensed data. A WBAN is an outstanding improvement in technology and its usage for the treatment of human beings is no less than a boon. It is embedded with IoT technology so the patient does not need to be in bed or in a hospital as happens currently in most circumstances [15].

However, the network challenges such as throughput, network delay, etc. cannot be ignored when using IoT technology for WBANs [16,17]. Because of physical size constraints, the sensors are equipped with very low power batteries [18]. These days, along with other limitations in WBANs such as buffer size, bandwidth, sensing power, etc. the most burning issue in this type of network is the availability of power for a long time [19]. In WBANs there are two types of data: normal data and emergency data [20]. To resolve all these issues the main focus currently is on the routing protocols used in WBANs. The routing techniques used in WBANs are proactive, reactive and clustering [21]. The traditional routing protocol is not a suitable solution for this type of network due to resource limitations [12]. A single-hop communication for data transmission is not considered a decent solution in WBANs. Instead, multi-hop data transmission works the best [20,21,22,23,24,25,26]. The existing solutions in WBANs for routing packets from source to destination have some limitations which we attempt to address in this research work in order to improve the efficiency further.

To solve the issues of energy limitation and WBANs’ lifetime enhancement a new scheme is proposed, named “Energy Harvested and Cooperative enable Routing Protocol” or EHCRP. In this protocol, energy harvesting is integrated inside the SNs to provide additional energy that enhances the network lifetime. Furthermore, link statistics are taken into consideration while forwarding the data to the next hop, which achieves better throughput and less end-to-end delay. This scheme works on the basis of multi-hop communication. The node with greater path cost estimation (PCE) will be nominated as data forwarder. In this solution, the data packets are divided into two types (normal and emergency). The path cost estimation function is used to calculate the cost of normal packets based on signal-to-noise ratio (SNR), total energy (TE), hop-count (HC), distance (d) from the central node coordinator (CNC) and node congestion level (NCL). The PCE technique balances the use of SN resources which increases the overall network performance. The second type of data packets (emergency data or life life-threatening data) is labeled as priority label. Each node in the network schedules data transmissions based on the priority label. Highest priority label packets are selected first for transmission. This is clearly depicted in the flowchart of the proposed scheme and also in Algorithm 1, 2 and 3. The presented technique includes:

(1) Link efficiency network model is presented which calculates the capability of the forwarder node in terms of its ability to send the received/sensed data. Link efficiency is based on four link quality parameters which are link quality indicator (LQI), packet reception ratio (PRR), SNR and received signal strength indicator (RSSI).
(2) The proposed protocol selects the forwarder node by calculating its PCE function derived from energy aware link efficiency of the selected node compared to non-selected ones.
(3) Supportive efforts communication has been used in which duplicated data is being discarded in successive transmission and not sent to CNC. In the proposed protocol the SN checks the sensed data for possible redundancy. If the sensed data (not in the case of critical situations) is similar to the prior sensed data, it is discarded otherwise it forwards the data to the sink.

**Algorithm 1: Data sensing and Topology discovery phase****Notation:****1.**HP = Hello Packet**2.**T.E = Total Energy**3.**L.E = Link Efficiency**4.**H.C = Number of hop-count between sink node (CNC) and leaf node (BSN)**5.**d = distance between SN to the sink CNC**6.**D = distance between leaf nodes**7.**(NT) = information included in Neighbor Table **8.**(HP) = information included in Hello Packet**Input: Hello Packets from neighboring nodes ‘i’ and ‘j’****9.**Start **10.**For each HP do**11.**   If HP (TE_i_, LE_i,j_, HC_i,CNC_, d_i,j_) = NT (TE_i_, LE_i,j_, HC_i,CNC_, d_i,j_)**Packet record in neighbor table for neighbor information****12.**E_j_ (NT)  
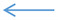
  TE (HP)**13.**LE_i,j_ (NT)  
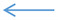
  LE_i,j_ (HP) **14.**   d_i,j_ (NT)  
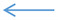
  d_i,j_ (HP)**15.**   hc_i,CNC_ (NT)  
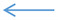
  HC_i,CNC_ (HP)**16.**   Else**17.**   Discard HP**18.**  If HP (TE_i_, LE_i,j_, HC_i,CNC_, d_i,j_) = null then**Add recored in neighbor table //new neighbor entry****19.**   TE_j_ (NT)  
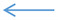
  TE_J_ (HP)**20.**   LE_i,j_ (NT)  
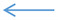
  LE_j_ (HP)**21.**   d_i,j_ (NT)  
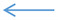
  d_i,j_ (HP)**22.**   HC_i, CNC_ (NT)  
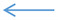
  HC_i, CNC_ (HP)**23.**
 **else go to line-10**
**24.**
     **end if**
**25.**
   **end if**
**26.****end for****27.**
 **End**



**Algorithm 2: Next Hop/Forwarder Node Selection phase**

***Notation:***

*1.*
N_i_ = Source Node
*2.*
NH_I_ = Next Hop for node N_i_
*3.*
CCN = Central Coordinator Node 
*4.*
NT = Neighbor Table
*5.*
SNR = Single to Noise Ratio between nodes
*6.*
PCE = Path Cost Estimation 
*7.*
RT = Routing Table
***Input: Records in Neighbor Table***

*8.*

**Start**

*9.*
    **If** N_i_ is at one hop to CNC then
*10.*
    send data directly to CNC
*11.*
    LE_i,j_ (NT)  
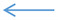
  LE_i,j_ (HP) 
*12.*

    **Else**

*13.*
    for each record in Neighbor Table 
*14.*
Calculate PCE = i=0Nα × 1R.E+ β × 1L.E+ γ ×HC+ δ ×d
*15.*
**If** (N_i_ PCE bit = 1) then // PCE previously checked = 1 not checked = 0
*16.*
 Select other neighbor node N_i_ from NT 
*17.*
   **else**

*18.*
    Compute SNR and ensure that it is heigheir than 1
*19.*
    Compute Neighbor distance d, HC, LE, and TE
*20.*
    TE = E_Current_ + E_Harvested_
*21.*
    If (N_i_ E_total_ < TH) then select other node from NT
*22.*
   **Else**
*23.*
    calculate PCE of N_i_
*24.*
    record PCE and update NT
*25.*
 Ensure all neighbors are checked
*26.*
    else send the data in single hop 
*27.*
 **else** skip sending and wait for another time
*28.*
    **end if**

*29.*
   **end if**

*30.*
  **end if**

*31.*

 **End**



**Algorithm 3: Cooperative Routing**

***Notation:***

*1.*
A_Data_ = Normal Data
*2.*
B_Data_ = Emergency Data
*3.*
D_packet_ = Data Packet
***Input: Data Packets from any node ‘i’***

*4.*

**Start**

*5.*
**If** B_data_ is emergent
*6.*
 Send it directly to CNC
*7.*
 **else**

*8.*
  send the data as normal A_data_
*9.*
   Otherwise discard the data packet // for redundant data
*10.*
    Discard D_packet_  // for redundant data
*11.*

**end if**

*12.*
 **End**


This research work is organized as follows: related research and literature is briefly reviewed in Section 2. The IoT-based WBAN along with energy harvesting is elaborated in Section 3, Section 4, elaborates the system model. The proposed system architecture is explained in Section 5. Section 6 gives details of decision-based cooperative-efforts. Section 7 explains the operational phase of the proposed protocol. Section 8 portrays evaluation and simulation, results and analysis with required figures and Section 9, presents the conclusions and suggestions for future work of this study.

## 2. Related Literature

The integration of WBANs and the IoT brings a new concept to the arena of sensor networks. WBANs fall under the WSN umbrella but differ as they have some unique characteristics. With the passage of time state-of-the-art techniques have been adopted to remove deficiencies in the internal working structure of WBANs. In different references numerous energy-efficient routing protocols have been anticipated for WBANs for diverse purposes such as thermal-conscious schemes, congestion control techniques and maximizing battery efficiency to extend the network lifetime. WBANs are mostly used in health-related applications that sense human body data, which in most cases is critical. Therefore, its timely transmission for further analysis to medical-related servers is of the utmost importance. Among all other technologies, routing is one of the main technologies, because the SNs’ sensed data needs to be forwarded to the medical-related server efficiently and in no time. Furthermore, due to resource constraints, such as limited battery capacity and transmission power due to the small size of the SNs, the network lifetime is compromised. Therefore, researchers nowadays are more focused on developing energy-efficient routing mechanisms along with other means of providing energy for WBANs. The aim of developing an energy-efficient routing mechanism is to enhance the network lifetime, network stability, and throughput and reduce end-to-end delay among others. A few state-of-the-art routing protocols are discussed below.

Rahat et al. [26], presented an efficient and reliable routing protocol for wireless body area sensor networks. It is claimed to be stable and efficient in terms of power consumption. A total of eight sensor nodes are deployed at different positions of human body which gather normal and critical data. Out of the total number of sensor nodes, two of the sensor nodes do not take part in multi-hop communication, but rather directly send data to the sink node. The remaining six sensor nodes forward the data to the best forwarder node, which is selected best on the calculated cost function. The parameters to select the best forwarder node are distance to the sink node and residual energy of the entire network. Extensive simulations are performed to prove the stable results.

Ullah et al. [27] presented a routing scheme known by the name “Energy-efficient Harvested-aware Clustering and cooperative-based routing protocol for WBAN (E-HARP)”. E-HARP is a multi-attribute-based harvested energy routing protocol, which takes different network-related parameters into consideration and selects an optimal forwarder node towards the sink node. In this scheme the sensed data is transmitted in case of need. It is duty of the cost function to select a cluster head (CH) that uses four main parameters such as SNR, residual energy, total energy and transmission power. The duplicated data is not forwarded by the SN to CH. Before transmitting the data forward, it is checked for its possible duplication with the previous rounds. Hence much of the network energy is saved by the removal of any duplicated data from the transmission pool.

Awan et al. [20] proposed a technique named Priority-based Congestion-avoidance Routing Protocol (PCRP). This algorithm is grounded on IoT technology, which is used for medical purposes. It uses multi-hop communication for data transmission and chooses a congestion-free path for quality of service (QoS) and emergency data to be forwarded for the purpose of increasing the efficiency. In this algorithm the authors have used a fitness function on the basis of residual energy (RE), node congestion level (NCL) and SNR. For highly important data they have supposed a priority bit.

Guangsong et al. [28], proposed an energy-efficient routing protocol for WBANs. In the proposed protocol the routing decision is based on the sensor node’s remaining energy, communication type, path-loss in the communication link and some other relevant parameters. It works based on three steps. In the first step, i.e., the initialization phase, a channel competition procedure is done. In the next step, i.e., the routing setup phase, routes having energy abundant are selected. Finally in the third step, time slots are assigned for data communication. Extensive simulations show that the proposed protocol achieved better results as compared to its counterpart in terms of minimizing the energy consumption along with efficiently utilizing the communication channel.

Zahid et al. [29], focused in their research work on two very important WBAN issues, prolonging the network lifespan and efficient communication. A complete novel scheme is proposed for WBANs named “Robust and Energy Harvested-aware Routing Protocol with Clustering Approach in Body Area Networks (EH-RCB)”. The authors targeted many WBAN network issues such as throughput, network lifespan, end to end delay, etc. They have proposed a system in which tiny sensors nodes are placed on the human body. These tiny sensors sense the important health-related parameters and forward it to two sink nodes. The two sink nodes are positioned on the front and back side of the human body. The highly critical data is sent directly to the sink node. Forwarder node selection is based on the optimal cost function (CF) value, which is calculated based on different parameters, such as residual energy, required transmission power, link SNR and distance from the sink node. Energy harvesting technique is adopted to provide additional energy to the sensor nodes in order to help out in prolonging the network lifetime.

Jitumani Sarma et al. [30] proposed a robust solution for WBANS. It works for one of the important health-related parameters, ECG. In order to save energy, the ECG signals are divided into acute and non-acute. The overall technical system is controlled and monitored by an IoT controller named Light Weight Power Management Controller.

Choudhary et al. [31], presented the “Energy Budget-based Multiple Attributes Decision Making Algorithm (EB-MADM)” which is designed to be low power, having a cluster-based routing mechanism. In this technique an optimal cluster head in each round is selected based on multiple factors, such as residual energy and low energy consumption. Furthermore, EB-MADM uses a cooperative effort which saves transmission energy by not sending redundant/duplicate data in consecutive rounds. The authors claimed that their simulation results shows better performance in terms of network stability, propagation delay, throughput and network lifetime as compared to its counterparts.

Mustaqim et al. [32], characterized an ultra-wide band (UWB) network for WBAN applications and all wearable IoT devices. They designed the UWB’s antenna using two unlike substrates, FR4 and denim textile material, which work from 2.9 to 11 GHz, as a result covering the whole UWB frequency band. A model of the proposed UWB antenna is then prepared to work on dissimilar antenna parameters such as gain, radiation patterns and reflection quantity (S11).

Sripada Soumya et al. [33], have focused on uses of the IoT for healthcare purposes. They worked on how to use biological devices to get human data and forward it to nursing institutes. They have defined special health services (SHS), to sense certain health parameters via wireless communication techniques such as WSN, RFID [6,11] and smart phones.

Rao Naveed Bin Rais et al. [34], proposed Fog-supported Internet of Things architecture for Remote Patient monitoring Systems using Wireless body Area Sensor Networks (WBASNs) protocol which works for a range of medical applications such as patient monitoring and their activities recognition. They integrated WBASNs with a communication structure for the purpose of getting the best and pre-defined results. They also used the model of fog computing in their proposed system. They claimed that the results and simulations showed much reduction of load on IoT WBANs and it worked much better.

Mallick et al. [35] proposed the so-called Energy-efficient Routing Protocol (EERP) scheme for wireless body area networks. The proposed scheme works to send the highly critical data to a medical expert by choosing the shortest path first. The selection of the shortest path is based on multiple network parameters. The authors have embedded their solution with many other algorithms such as a travelling salesman approach and ant colony optimization (ACO). For the extension of network lifespan, they used a Bayesian game formulation. The critical data is directly routed to medical experts without any delay. The proposed scheme is claimed to achieve better performance in terms of network lifetime, throughput and reduced end-to-end delay.

## 3. IoT-WBAN with Energy Harvesting

**Figure 2:**
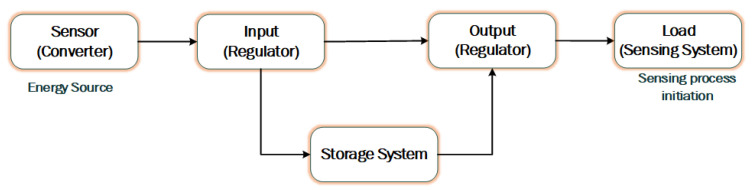
Energy harvesting system with components architecture.

Energy harvesting is the process by which a SN can power itself [36,37,38,39,40,41,42] as shown in Figure 2. The SNs can be equipped with many types of techniques which help the node produce energy from the environment automatically. A summary of energy harvesting sources and the used mechanisms is shown in Table 1. Due to the small size of the SN, the available resources in it is also limited, such as small batteries having less available energy. The initial energy of the SN is 0.5 Joule which is very small. Due to this limitation, the energy available for the operation of the SN, such as transmission and reception of data, is limited [43,44,45,46,47,48,49,50,51]. In order to use a WBAN, practical efforts have been made worldwide to resolve problem of supplying energy to SNs through reliable and efficient techniques. The efficiency of power supply is ensured during the design of an integrated circuit; the capacitance provided is a critical aspect which is used for the regulation of operating clock frequency [44]. It has great effects on the energy depletion of the circuit. A high clock rate means more power usage. It is therefore recommended to design a low clock frequency circuit as shown in Equation (1).

(1) R=K·V

where, *R* represents the total quantity of charge, *K* represents the magnitude of capacitance, and *V* represents the current of the capacitor. The capacitor current is directly proportional to the quickness of the voltage change inside the capacitor. The voltage of the capacitor rises if the current is harvested during input mode. The integrated circuit (IC) should check the voltage more often in cases where the voltage of the capacitor changes rapidly.

## 4. System Model

The proposed System Model is categorized into network model and energy consumption model, which is elaborated below.

### 4.1. Network Model

**Figure 3:**
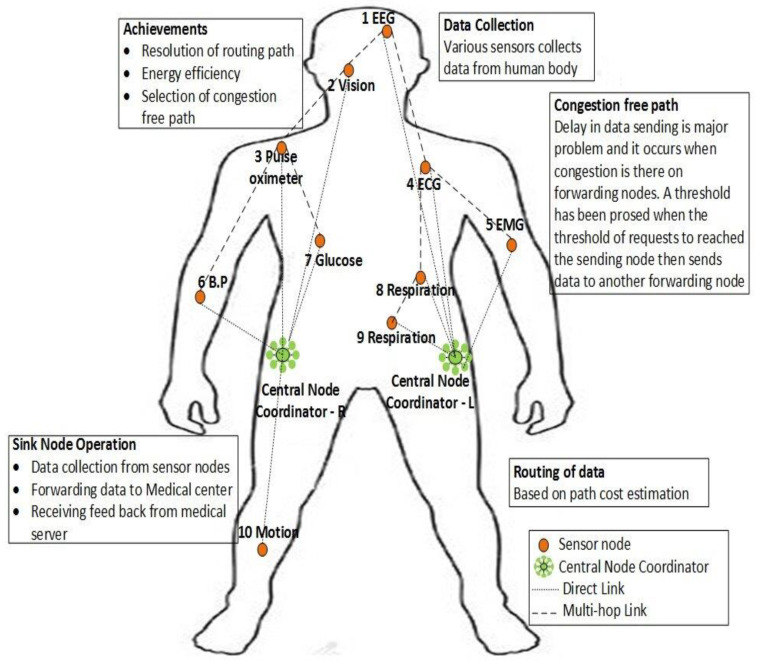
Node deployment in EHCRP.

The proposed IoT-WBAN network consists of ten (10) heterogeneous SNs having limited hardware resources. These SN’s are deployed in/on different parts of the front and back side of human body. Beside these SNs two CNCs are also deployed on the left and right hips. The position of the CNCs is important in order to minimize line-of-site (LoS) problems which can affect network connectivity. The CNC simply receives data from SNs and forwards it to a personal digital assistant (PDA) for further processing. Table 2 provides a detailed description of the SNs and CNCs. The deployment of different health-related SNs on different parts of the human body has been portrayed in Figure 3.

The communication scenario is in single-hop and multi-hop. Single-hop takes place when the sensed data is either serious or SN is near to CNCs. If the scenario is different as mentioned above, then the SNs communicate its data using multi-hop communication using the Path Cost Estimation (*P.C.E.*) function. The required factors for *P.C.E.* computation are calculated by SNs and then send to CNC. This is performed after assigning predefined time slots to each SN at initialization stage of each sensing/transmission round by using TDMA technique.

The following assumptions have been made in proposed research work:

(1) Due to mobility of human body, the positions of the SNs may frequently change.
(2) Power consumption during computation/processing is considered to be negligible. As its low compare to data packet transmission or reception.
(3) To cater limited energy availability problem, an energy harvesting scheme is used in the proposed technique. With the passage of time SNs could harvest energy (as shown in Table 2) to prolong network lifetime. The prediction of the harvested energy of each SN is articulated in Equation (2):

(2) $$E_{\mathrm{Harv}}\;(t,\;Q_{\mathrm{Setup}})\;=\;\int_{t}^{t+Q_{setup}}\lambda_{i}d\tau$$

where *E_Harv_* represents probable harvested energy by a SN *i* in a pre-defined period τ. The term $\lambda_{i}\left(\tau \right)$ represents the charging rates of SN *i* in time τ.

(4) Ten sensors are positioned in/on human body of which 07 are wearable and 03 are implanted. Two CNCs have been deployed to minimize Line-of-Sight (LoS) problem.
(5) The IEEE 802.15.6 BAN channel modeling project [18] is used for path loss. It is shown in Equation (3):

(3) Pl (f, d)=Pl (f)×Pl (d)

where Pl (f) and Pl (d) represent path loss due to frequency and distance.

(6) The Euclidean distance formula equation is used for the calculation of distance among SNs and Sink Node. The path loss also effects on distance which necessary to be considered while computing distance as shown in Equation (4):

(4) P.Ldb(d)=PL0(db)+10ηdd0

where P.L and $PL_{0}$ represent the path loss and path loss at a specific distance *d_0_* (reference distance). η is path loss exponent/constant.

### 4.2. Energy Consumption Model

The consumption of energy by the SNs during the transmission and reception of data packets are estimated via a first order ratio model [28]. Equation (5), states the usage of energy during transmission of *L* bits to the destination receiver SN at distance *D*:

(5) $$E.T\;(L,Di,j)\;=\;L\times E_{Tran}+L\;\times E_{amplifier}+\;D_{i,j}^{2}$$

where *E.T* represents energy consumption during data transmission, *L* represents the bits/packet and *D* represents the distance from sending sensor to the receiver sensor, *E_amplifier_* represents the energy spent by the electric circuit. The energy consumption to receive *L* bits of data is expressed in Equation (6):

(6) $$E.R\;(L,D_{i,j})\;=\;L\;\times \;E_{recp}$$

where *E.R* represents energy consumption during data reception, *L* represents the length of bits/packet received by a sensor and *E_recp_* is the energy consumed during reception by the receiver circuitry, *Di j* is the distance between sensing SN and receiving SN.

Furthermore, Equation (7) represents the consumption of energy during aggregation of data:

(7) $$E.A=L\;\times V_{supply}I_{0}V_{supp}/K_{p}V_{t}M_{cycle}$$

where *E.A* represent the energy aggregation, *I_0_* denotes the current loss, *K_P_* represents constant and its value depends on processor type, *V_t_* represents the transmission voltage, *M_cycle_* denotes the cycle of the machine or CPU spent during cycle.

The SNs deployed/attached on human body senses for physiological data of human body. Energy spent during this process is referred as energy sensing (*E.S*), which is represented in Equation (8):

(8) $$E.S=L\;\times \;\left(V_{sen}+\;I_{sen}+\;T_{sen}\right)$$

where *L* is the packets of bits and *V* is the voltage supplied during sensing, *I* represent the actual present consumed and *T* represent the time lapsed during sensing.

The energy consumed during a cycle span of bit transmission could be calculated and is represented in Equation (9):

(9) $$E_{\mathrm{consumed}}\;=\;E.S+\;E.A+\;E.T+\;E.R$$

The remaining energy after the completion of all types of processing is considered as residual energy which is represented in Equation (10):

(10) $$E_{\mathrm{residual}}=E_{total}-\;E_{consumed}$$

## 5. Energy Harvested and Cooperative-Enabled Routing Protocol (EHCRP) Scheme

In the proposed work, more focus is given to solve mainly the following issues:

- Network stability/network lifetime; which is always remains one of the main issues in WBAN from very beginning. The issue arises due to the small size and limited battery backup in the tiny SNs.
- Congestion on forwarder nodes.
- Line of sight (LoS) which causes path loss and end to end delay in WBAN because of different body postures of humans during mobility. For the purpose dual coordinator nodes concept has been used which are deployed on left and right hips of the human body.
- Transmission of duplicated data causes more energy consumption. It is eliminated in EHCRP tremendously.

### 5.1. Sensor Nodes Deployment Phase

Deployment of all SNs along with coordinator nodes is the very first phase of WBAN. Total ten (10) nodes having eight (08) SNs and two (02) coordinator nodes are position on/in various location of human body as exposed in Figure 3, also described in Table 2. By using RSSI, the SNs and both the coordinator nodes start to calculate their location and distance from their neighbors and the coordinators node. After distance calculation all SNs including along coordinators transmit a BEACON message in network. The BEACON message includes of Node ID, distance from its neighbor (d), destination node ID, and Residual Energy (*R.E*) and node location.

#### Path Cost Estimation

Most of the existing protocols attempt to select the shortest path from/to the sink/coordinator, but this research work additionally considers link efficacy for the selection of next hop for data communication. This approach presents a path cost estimation (*P.C.E.*) technique work on total energy (*TE*), hop-count (*HC*), link efficiency (*LE*), distance (*d*) and the node congestion level (*NCL*) of a node to the CCN as shown in Equation (11). The least value of PCE is used to choose next-hop as a forwarder node:

(11) $$P.C.E\;=\;\frac{\left(\alpha \;\times R.E+\beta \times L.E+\gamma \times HC+\delta \times d+\omega \times NCL\right)}{SNR}$$

where *α, β,* γ, and δ denote weighting factors for *TE, LE, HC* and *d*, respectively, while considering signal to noise ratio as well.

Based on the exchange of BEACON messages among SNs, the path-loss is calculated. If the path-loss is on the higher side, the network quality is considered as poor, alternatively if the path loss is lower, the quality is considered as good. Considering our energy-centric communication model development for WBAN, we employ the simplistic path-loss model.

To select the optimal forwarder node, distance is an important factor to be considered. If the location of CNC is *X*_0_ and *Y*_0_ axis of central node and the location of source node is *X*_1_ and *Y*_1_ axis of SN, then the distance of SNs from central node can be calculated through the Euclidian distance as given in Equation (12):

(12) $$d_{\left(\mathrm{sink},\;\mathrm{SN}\right)}\;=\;\sqrt{{\left(X_{0}-\;X1\right)}^{2}+\;{\left(Y_{0}-\;Y_{1}\right)}^{2}}$$

Another most important parameter in network is Link efficiency *L.E* that directly effects the energy consumption, which is shown in Equation (13). Quality of service (QoS) of a network is mainly based on *L.E* which ensures maximum end-to-end transmission:

(13) $$L.E_{\;i,\;j}=\sum \frac{PR_{n}}{PS_{s}}$$

where *L.E* denotes link efficacy between nodes *i* and *j* through *PR_n,_* i.e., the total packets received at neighbor node and *PS_s_*_,_ the total packets sent from source node. If the *L.E* is low, it may cause packet loss during transmission.

Each SN sends complete information in a REPLY message to the CNC after important estimation. The REPLY message comprises of node ID, total energy, possible network energy loss, and required transmission power T.P and other information.

In some cases, noise exists in paths which create major problems in data transmission. WBANs might be interacting with other communication technologies such as Zigbee, Bluetooth WPAN, WLAN, cellular system etc. for data transmission. Because of the interference of these technologies the signal strength may decrease in the WBAN. If the noise ratio gets higher, the signal strength will be low and can lead to data packet loss, on the other hand if the noise ratio becomes low the signal strength is higher, leading to minimum data loss of packet. SNR can be computed by Equation (14):

(14) $${\mathrm{SNR}}_{\mathrm{Reciever}}\;=\;P_{Trans}-\;Pl\left(d\right)-\;N_{P}$$

where, *SNR_Reciver_* is ratio of snr at receiver end, *P_Trans_* is transmission power, *Pl (d)* represents path loss at distance *d* and *N_P_* represents noise power.

In order to get the optimal results from any network, the node congestion level (NCL) must be properly controlled. Congestion is the event in which the data rate exceeds the available capacity at any point in WBAN [1]. It is categorized as node level congestion and link level congestion in WBANs. In this research work, more focus is on node level congestion. Node level congestion can be calculated using Equation (15):

(15) $$\mathrm{NCL}(\mathrm{Current}\;\mathrm{Queue}\;\mathrm{Size})\;=\;Q_{total}-\;Q_{free}$$

where *Q_total_* represents total buffer space of the sensor and *Q_free_* represents free available buffer space.

### 5.2. Topology Formation Phase

**Figure 4:**
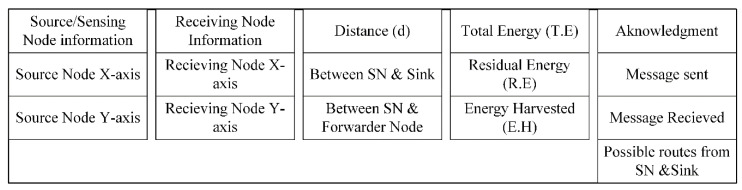
Hello Packet block diagram.

Once all the nodes are deployed, and path cost estimation is done, the SNs then exchange hello and reply packets. They exchange messages comprising different factors, such as node location, node ID, total energy and distance to/from the sink and other neighboring nodes. After exchange of a hello packet (HP), each node retains a neighbor table (NT) that further helps in updating the routing table of each node. Hello packets are broadcast during the topology formation phase by the CNC to all neighboring nodes. The nodes receive the packet and give a reply as acknowledgment to the CNC. The CNC also updates its routing table with the BSNs information received in the form of feedback messages. The header included in a normal hello packet is shown in Figure 4 [43,46]. These headers are included in a normal hello packet as well as in normal data packets, but in emergency data transmission, an emergency label is added to the start of the packet. By reading the first label as an emergency, the data packet is given the highest priority and the data is sent directly to the CNC. If the hello packet is sent to the neighbor which does not send it in a specific time, then the link is considered as broken. Information about CNC and all possible routes are also exchanged during this process. The total energy (TE) used during the phenomena comprises of residual energy and harvested energy.

### 5.3. Scheduling Phase

When all sensors nodes are registered with the CNC, then they need to be schedule for normal communication. In this stage the CNC assigns time slot to each SN. The time slots are provided considering for either normal or emergency data transmission. Where the later gets a special certain time slot in Contention Free Period (CFP) from MAC super frame. This ensures quick and efficient transfer of emergency data. CNC doesn’t sense its own data but collects data from SNs and forward it to PDA.

### 5.4. Forwarder Node Selection

The priority of a sender node is to send data by involving lesser number of hops counts to the CNC. However, for the sake of energy balancing the path with more number of hops counts may also be considered.

## 6. Data Transmission through Decision-Based Cooperative Effort Number Already Used

When the sensor senses data, it needs to be forwarded to sink node. While transmitting the data first the SN checks its total current energy. If the available energy is found to be lower than the pre-defined threshold, then the sensor has to wait for the next round of data transmission. Meanwhile it harvests energy for the upcoming round.

Sensors may sense the same data in a WBAN in repetitive data sensing rounds. Sensing this duplicate data consumes valuable sensor energy. Much of the sensor’s energy can be saved by avoiding duplicate data during sensing or transmission. This can be achieved by using a supportive decision-based data transmission scheme as shown in Algorithm 3. In the EHCRP protocol, the duplicate sensed data in consecutive rounds is eliminated from the transmission pool. Before deciding to send the data to a sink node, a minimal process is done to check whether to send the data or not. Data transmission takes place if either of the following conditions is fulfilled:

(i) The data which is transmitted to sink node is not similar to the data stored in former round.
(ii) If the data is of an emergency/critical nature.

## 7. Operational Phase of the Proposed Protocol

**Figure 5:**
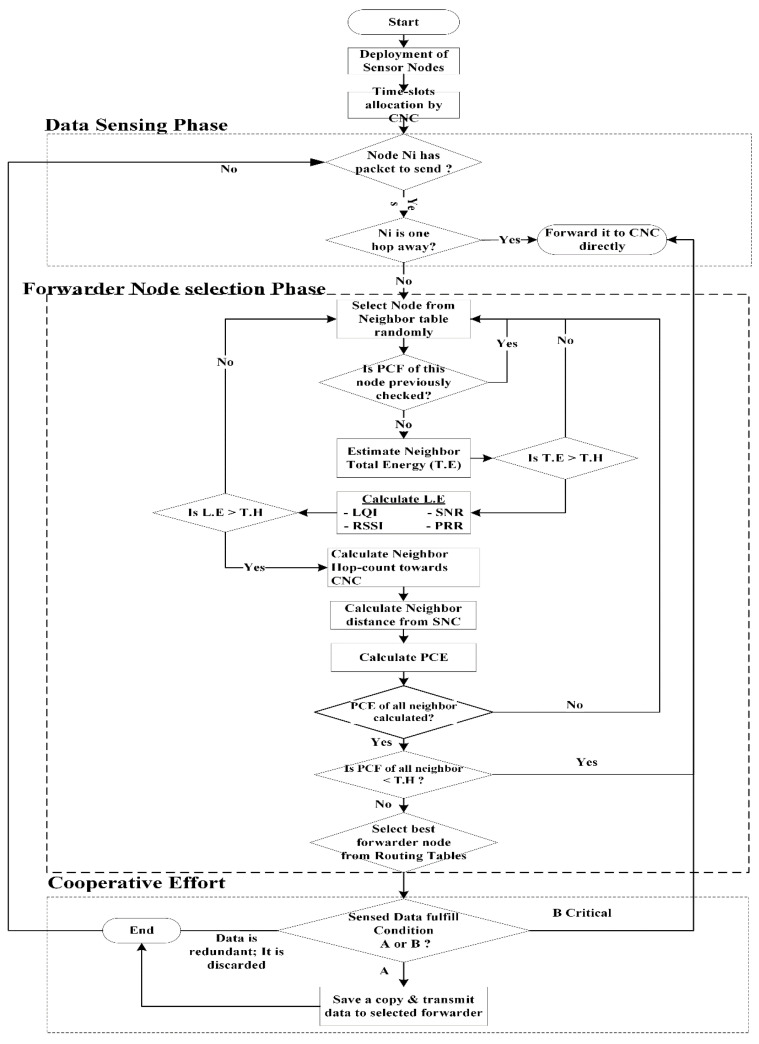
Flowchart of EHCRP.

The overall routing phases of EHCRP have been divided into three stages: i.e., data initialization and sensing phase, forwarder node selection phase and cooperative effort phase which are represented with the help of the flow chart shown in Figure 5.

## 8. Performance Evaluation and Simulation

The performance evaluation of the proposed scheme is paralleled with four different routing techniques called as E-HARP [27], PCRP [20], EERP [35] and EB-MADM [31] by using the NS–2 simulator. For the comparison, the unit of time is taken in rounds. In a single round each sensor senses a data packet and forwards it ahead. The total time is measured between the first round and before finishing the last round. This scheme is checked with other contemporary methods in terms of residual energy, network lifetime, stability, end-to-end delay and network throughput.

The topology used for proposed WBAN scheme has a total of ten SNs and two sink nodes, called the central node coordinator (CNC). These nodes are placed on various parts of the human body, as shown with details in Figure 3. These deployed tiny sensors sense human-related medical data such as glucose, BP, EMG, ECG and other important medical parameters. These sensed data are then forwarded to the CNC for further required processing. Details of the simulation are given below in Table 3.

### 8.1. Network Lifetime

**Figure 6:**
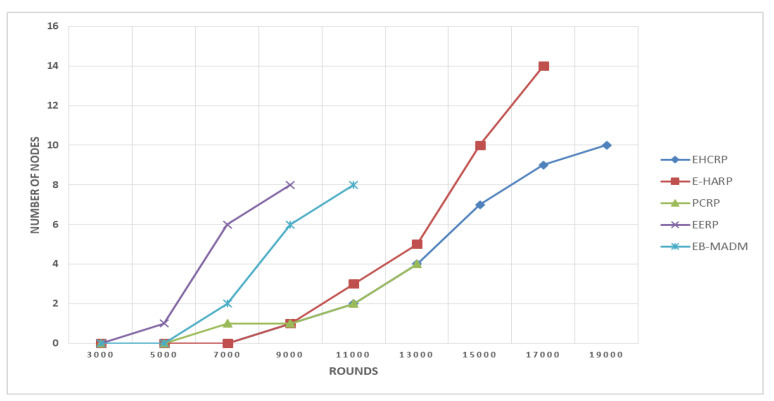
Breakdown of network lifetime.

The total time span of any network is referred to as the network lifetime. This is considered one of the main judging criteria of any kind of sensor network. As a WBAN is one of the prominent types of WSN the same criterion is also applied here. The proposed scheme is submitted to a network lifetime test to check its efficacy. The comparisons of the proposed scheme with other contemporary techniques in terms of network lifetime can be seen in the Table 4. It is also shown in Figure 6. These results show the supremacy of the proposed scheme. According to these simulations results, the proposed scheme went beyond round 17000th, where in the other schemes the network lasted till 17000th rounds. Only, E-HARP was the closet contester of EHCRP in this department while EERP is far off in this race. EB-MADM also showed good performance in this regard, as it lasted till 10000th round. The better performance of proposed and E-HARP schemes in term of network lifespan is due to implantation of smart harvesting technique and energy efficient routing in both routing protocols.

### 8.2. Stability Period

**Figure 7:**
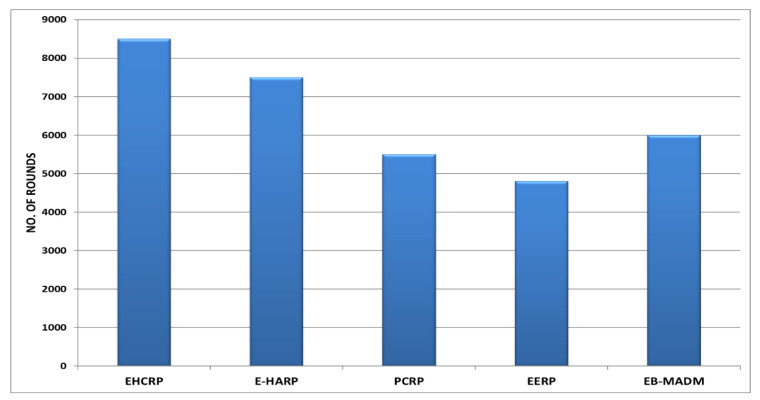
Breakdown of Network Stability (First-Node-Death).

**Figure 8:**
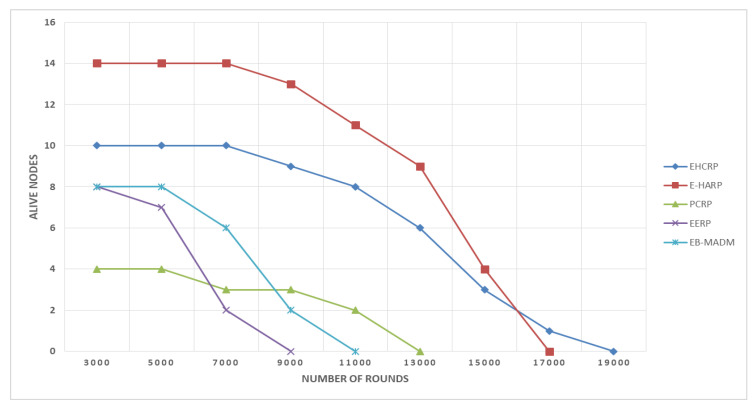
Breakdown of Live Nodes vs. Rounds.

This is defined as the total period of time till death of first node of a network or while all sensors are active and alive. This parameter is also considered important in the efficiency of any type of WSN network including WBANs. The proposed scheme worked well in this regard compared to other protocols. The results shown in Figure 7 and Figure 8 are evidence of this. Figure 7 and Table 5 show that the first node of EHCRP protocol died at the 8500th round, which is much higher than the 7500th round of E-HARP, 5500th round of PCRP, 4800th round of EERP and 6000th round of EB-MADM, respectively. It is clear from Figure 7 that the proposed system delays the death of the first node, which means it performed better than others. The high network stability of the proposed algorithm is due to the smart harvesting energy units in each node and the elimination of extra processing overload due to duplicate data transmission.

### 8.3. Residual Energy

**Figure 9:**
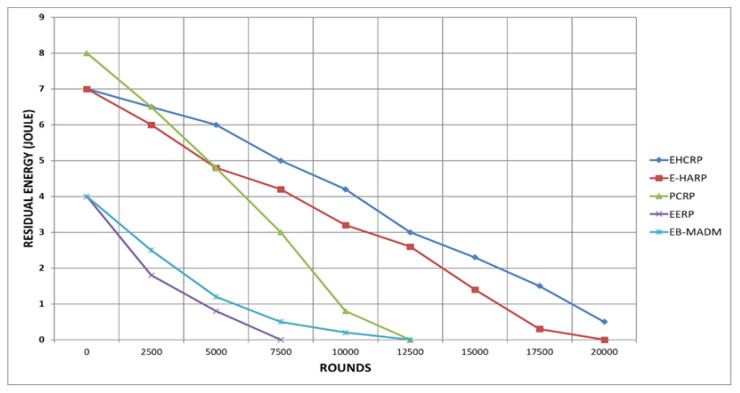
Breakdown of Energy Consumption.

Residual energy is defined as the remaining energy of any SN in the deployed sensor network. The energy of the SNs is consumed by different operation such as sensing data, storing it, receiving and forwarding, processing etc. The energy consumption is burning parameters for any network having battery operated devices such as in WBANs. This presented scheme shown good performance in terms of energy usages which is portrayed in Figure 9 and in Table 6. EHCRP is leading this branch compared with other schemes. The energy depletion process of EHCRP and E-HARP during simulation was almost equal in ratio. The proposed protocol energy lasted almost till 20000th round. The energy of EERP depleted very quickly and it completely drained before 7500th round, which shows bad performance. Also, PCRP didn’t execute well with respect to residual energy depletion as its energy lasted only to 10000th round. The main reason behind the better performance of EHRCP in terms of less energy consumption is its optimized working mechanism.

### 8.4. Throughput

**Figure 10:**
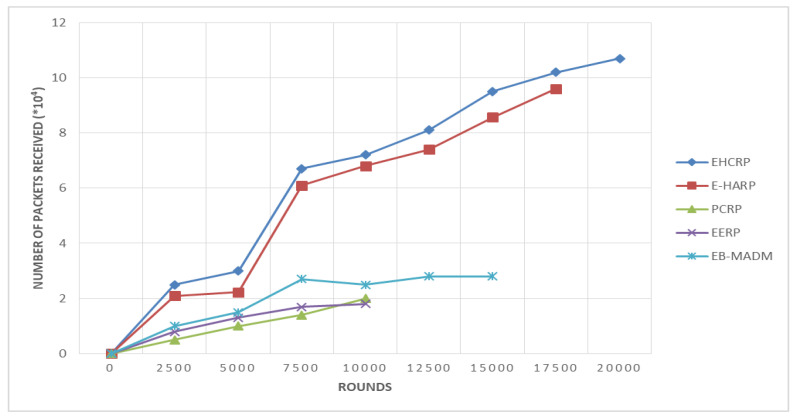
Breakdown of network throughput.

It is fruitful to transmit data from SN to the SN or sink/coordinator node in a given time. More throughputs per unit time are considered a good feature for any kind of WSN, especially WBANs. It is the successful delivery of packets in data transmission. Throughput is one of the most important parameters while judging any scheme or protocol for data transmission. High throughput means better performance. The results in Table 7 and Figure 10 show the success of EHCRP in this aspect. At the initial stage of the throughput simulation, the fluctuations among the compared protocols were minimum but after the 7500th round, it increased with a distinct margin. The reason is that at round 7500 the throughput of EHCRP becomes more than double (6.7) compared to the 5000th round, which was 3. Overall the proposed protocol achieved 10.7 × 10^4^ in 20,000 s, whereas its counterparts, did not exceeded 4.7 × 10^4^, except for E-HARP which was the closest and achieved 9.6 × 10^4^ throughput. The main reason behind the better performance of EHCRP in terms of throughput is that it considers different parameters of the communication link before the transmission, such as SNR, link quality, etc. This ultimately avoids the loss of data packets during transmission.

### 8.5. End-To-End Delay

**Figure 11:**
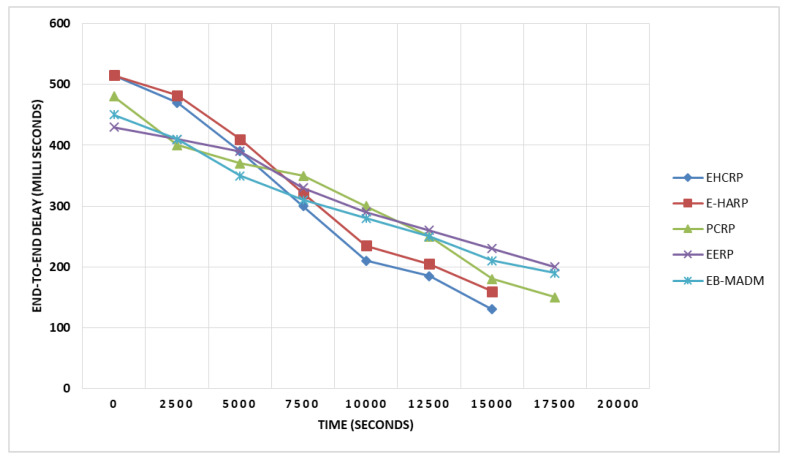
Breakdown of end-to-end delay.

This is the time taken by a transmitted packet to go from source to destination. As WBANs are used to monitor important parameters related to human’s health, the end-to-end delay must be incredibly small to avoid any mishap. In WBANs the sensed data should move quickly to the target destination. Considering the significance and importance of end-to-end delay in WBANs, this work aimed to decrease it to a minimum level. The results of end-to-end simulation are shown in Table 8 and in Figure 11. The results demonstrate that the EHCRP protocol performs best in terms of end-to-end delay. The dual sink concept plays an important role in minimizing end-to-end delay. At the initial stage, the performance of EHCRP was almost equal to the other counterparts but later on it improved compared to its peers. At the initial stage during initialization the proposed model does a massive calculation by transmitting beacon messages due of which it performed the same as others, but after the 7500th round, the end-to-end delay decreased distinctly in EHCRP and came down to 130 ms whereas its counterparts have more delay in communication.

## 9. Conclusions and Future Work

In this research work, the EHCRP protocol which involves using tiny sensors for proficient selection of the next hop node in WBANs is proposed. Prolonged network lifespan and proficient emergency data delivery within given time constraints are the two major necessities for normal functioning of WBANs. EHCRP tries to achieve both these important goals. The most packets with life-threatening data are listed before the normal data packets during transmission. Also, interference is one of the bottleneck factors that significantly affect WBAN performance. Therefore, in this research work we tried to choose a path for data delivery which is less congested or free for traffic. The achieved results are evaluated by comparison with state-of-the-art routing techniques i.e., E-HARP, EB-MADM, PCRP and EERP. The results shown in the tables and figures in the “Performance and Evaluation” section clearly attested to the fact that the proposed protocol outclassed all other contemporary techniques. The E-HARP routing technique was the closest competitor of the proposed scheme in terms of all parameters considered in the “Performance and Evaluation” section. The high performance of the proposed scheme is due to smart harvesting system and dropping of redundant data packets from the transmission pool. As a future work, the intention is to extend the proposed work with more WBAN link quality metrics along with authentication security. Thus with an efficient routing link in each situation a patient’s data may only be checked by his/her own medical practitioner or caregiver.

## Figures and Tables

**Table 1:** Energy harvesting sources and their mechanisms [30].

A Summary of Energy Harvesting Source, Mechanism and Efficiency				
Source	Sensing	Source Type	Harvested Power [30,37]	Advantages
Solar	Photovoltaic Cells	Natural	12 mW/cm^2^	Inexhaustible and clean
Ambient Indoor light	Photovoltaic Cells	Artificial/Natural	100 mW/cm^2^	Inexhaustible and clean
Thermoelectric	Thermocouple	Artificial	60 μW/cm^2^ at ΔT = 5 °C	Small size, no vibration and noise and reliable performance
Ambient Air flow	MEMS turbine	Natural	1 mW/cm^3^ at 301/min	-
Wind	Anemometer	Artificial/Natural	Upto 1200 mW/day	Widely available
Footfalls	Piezoelectric	Artificial	5 W	Easily available
Finger motion	Piezoelectric	Artificial	2.1 mW	High conversion efficiency
Exhalation	Breath Mask	Artificial	0.4 W	High conversion efficiency
Breathing	Ratchet-Flywheel	Artificial	0.42 W	Pollution free energy
Blood Pressure	Micro-generator	Natural	0.37 W	-

**Table 2:** Biological SNs details with SNs IDs [30].

Node ID	Location on the Body	Health Parameter	Data Rate (kbps)	Bandwidth (Hz)	Type	Power Consumption [30]
1	Head	EEG Sensor	43.2	0–150	Wearable	Low
2	Right shoulder	Pulse Oximeter Sensor	10–16	0–1	Wearable	High
3	Chest	ECG Sensor	288	100–1000	Implanted	Low
4	Abdomen left	Lactic acid Sensor	400	0–100	Implanted	Low
5	Face	Vision sensor	260	100–1000	Wearable	High
6	Left Arm	EMG Sensor	320	0–10,000	Wearable	Low
7	Abdomen right	Glucose Sensor	1600	0–50	Implanted	Extremely Low
8	Left Hip	Respiration	320	0–10,000	Wearable	Low
9	Right arm	Blood Pressure Sensor	10–16	0–1	Wearable	High
10	Right foot	Motion Sensor	35	0–500	Wearable	High
11	Left U-Hip	Central Node Controller–L	4Gbps	-	Wearable	High
12	Right U-Hip	Central Node Controller–R	4Gbps	-	Wearable	High

**Table 3:** EHCRP simulation constraints.

Parameters	Values
Simulation area	2 × 2 m^2^
Total SNs	10
Number of SN nodes	02
Sensor’s Position and CNCs	Refer to Table 2
E_initial_	0.5 J
E.T	16.8 nJ/bit
E.R	36.2 nJ/bit
E.A	2 nJ/bit
Wavelength (λ)	0.138m
Frequency (f)	2.4 GHz
Payload	3000 bits

**Table 4:** Lifeless Nodes vs. Rounds.

Protocols	1st Node Dies at	No. of Dead Nodes at Different No. of Rounds								
		3000	5000	7000	9000	11,000	13,000	15,000	17,000	19,000
EHCRP (Proposed)	8500	0	0	0	1	2	4	7	9	10
E-HARP	7500	0	0	0	1	3	5	10	14	-
PCRP	5500	0	0	1	1	2	4	-	-	-
EERP	4800	0	1	6	8	-	-	-	-	-
EB-MADM	6000	0	0	2	6	8	-	-	-	-

**Table 5:** Live nodes vs. rounds.

Protocol Name	No. of Alive Nodes at Different No. of Rounds								
	3000	5000	7000	9000	11,000	13,000	15,000	17,000	19,000
EHCRP (Proposed)	10	10	10	9	8	6	3	1	0
E-HARP	14	14	14	13	11	9	4	0	-
PCRP	4	4	3	3	2	0	-	-	-
EERP	8	7	2	0	-	-	-	-	-
EB-MADM	8	8	6	2	0	-	-	-	-

**Table 6:** Residual Energy vs. Time (s).

Protocol Name	Residual Energy (J)								
	0	2500	5000	7500	10,000	12,500	15,000	17,500	20,000
EHCRP (Proposed)	7	6.5	6	5	4.2	3	2.3	1.5	0.5
E-HARP	7	6	4.8	4.2	3.2	2.6	1.4	0.3	0
PCRP	8	6.5	4.8	3	0.8	0	-	-	-
EERP	4	1.8	0.8	0	-	-	-	-	-
EB-MADM	4	2.5	1.2	0.5	0.2	0	-	-	-

**Table 7:** Network throughput vs. time (s).

Protocol Name	Packets Received at Different Interval of Time								
	0	2500	5000	7500	10,000	12,500	15,000	17,500	20,000
EHCRP (Proposed)	0	2.5	3	6.7	7.2	8.1	9.5	10.2	10.7
E-HARP	0	2.1	2.23	6.1	6.8	7.4	8.56	9.6	-
PCRP	0	0.5	1	1.4	2	2.5	4	4.6	-
EERP	0	0.8	1.3	1.7	1.8	2	2.8	3.9	-
EB-MADM	0	1	1.5	2.3	2.5	2.8	2.8	-	-

**Table 8:** End-to-End delay vs. time(s).

Protocol Name	End-to-End Delay (ms)								
	0	2500	5000	7500	10,000	12,500	15,000	17,500	20,000
EHCRP (Proposed)	515	470	390	300	210	185	130	-	-
E-HARP	515	482	410	321	235	205	160	-	-
PCRP	480	400	370	350	300	250	180	150	-
EERP	430	410	390	330	290	260	230	200	-
EB-MADM	450	410	350	310	280	250	210	190	-
